# Little Evidence for Genetic Susceptibility to Influenza A (H5N1) from Family Clustering Data

**DOI:** 10.3201/eid1307.061538

**Published:** 2007-07

**Authors:** Virginia E. Pitzer, Sonja J. Olsen, Carl T. Bergstrom, Scott F. Dowell, Marc Lipsitch

**Affiliations:** * Harvard School of Public Health, Boston, Massachusetts, USA; † Centers for Disease Control and Prevention, Atlanta, Georgia, USA; ‡ University of Washington, Seattle, Washington, USA

**Keywords:** avian influenza, genetic susceptibility, models, binomial, disease clustering, dispatch

## Abstract

The apparent clustering of human cases of influenza A (H5N1) among blood relatives has been considered as evidence of genetic variation in susceptibility. We show that, by chance alone, a high proportion of clusters are expected to be limited to blood relatives when infection is a rare event.

Since December, 2003, 36 family clusters among 261 confirmed human cases of influenza A (H5N1) have been documented (*1**,**2*). These clusters range in size from 2 to 8 infected persons; in only 4 clusters were 2 unrelated family members (e.g., husband and wife) infected. This pattern has been considered by the World Health Organization as evidence of genetic variation in susceptibility (*3**–**5*), but we show this observation provides little grounds for this inference. We describe a null model in which nuclear families experience a common exposure to an avian influenza virus. The observed degree of clustering in blood relatives is consistent with that expected by chance alone in the absence of genetic variation in susceptibility; other features of the data are also consistent with the null model.

Our model assumes all persons are equally susceptible, such that they have the same probability of infection, τ, and ignores possible human-to-human transmission (see Technical Appendix). The number of infected family members follows a binomial distribution with mean *nτ*, where *n* is the number of exposed persons in each family. A cluster is defined as a family in which >1 person is infected; clusters are limited to blood relatives unless both parents are infected.

We compare our model to the observation that 32 of 36 clusters that occurred from December 2003 to December 2006 consisted only of blood relatives (*p_B_* = 0.89, 95% confidence interval 0.74–0.97; Table in Technical Appendix). When the probability of infection is low, most clusters consist of 2 infected family members, and by simple combinatorics, these 2 are usually blood relatives, which is consistent with the observed data (Figure 1).

**Figure 1:**
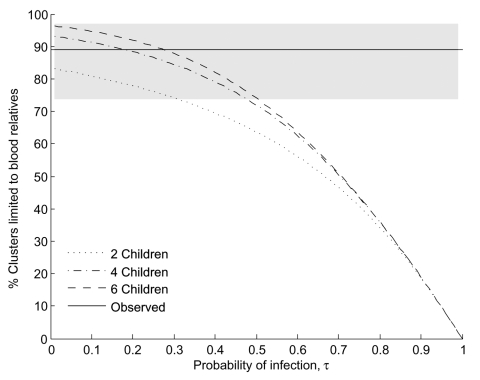
Proportion of clusters limited to blood relatives versus the probability of infection (τ) under the null hypothesis (no variation in susceptibility). Point estimate of the observed data is represented by the solid black line; the shaded region represents the 95% confidence interval.

For a given a nuclear family size, the null model also predicts the proportion of all cases that are part of a cluster and the average number of cases per cluster. Neither of these measures follows a simple distribution; we therefore use simulated data to determine what ranges of our parameters (τ and *n*) are consistent with the observed degree of clustering both in families and among blood relatives. We estimate the mean and 95% prediction intervals for the proportion of cases occurring in clusters when there are 261 cases, and for the average number of cases per cluster when there are 36 clusters. The expected proportion of cases occurring in clusters is similar to the observed data when the probability of infection is low (τ<0.15) (Figure 2). The observed average number of cases per cluster, however, is consistent with slightly higher probabilities of infection, larger family sizes, or both (Figure 2).

**Figure 2:**
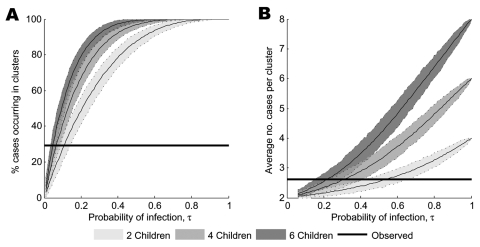
Relationship between data simulated under the null model and the observed pattern of family clustering for A) the proportion of cases occurring in clusters (given 261 total cases) and B) the average number of cases per cluster (given 36 clusters). Estimates of the mean are represented by solid lines; the shaded regions between the dotted lines show 95% prediction intervals for 1,000 simulations. The observed data are represented by the solid black lines.

The discrepancy between the number of cases per cluster and the proportion of cases in clusters may be due to between-family variation in τ. If the probability of infection is low for members of most exposed families and higher for members of a few exposed families, then most cases may come from families in which τ is low, but most of the clusters will occur among families for which τ was higher. This will lead to a lower proportion of cases occurring in clusters and a higher average number of cases per cluster, as is observed. Although it is possible that such variation may be genetic, it could also result from between-household heterogeneity in intensity of exposure to infected birds (or intensity of shedding in birds to which different households are exposed), household hygiene, living conditions, and the like. Human-to-human transmission of the virus could also lead to larger than expected cluster sizes because having >1 case(s) within a family would increase the risk of subsequent cases occurring, and it could not be ruled out in several clusters (*6**,**7*).

Qualitatively, the data suggest the existence of nongenetic, between-household variation in risk. If such nongenetic variation were absent, then in any given village, nearly all pairs of cases occurring among unrelated persons in the same village would be in different households. Roughly, the chance that a pair of cases in unrelated persons in a village would be from the same household as opposed to different households would be 1/*H*, where *H* is the number of households in a village. With 4 pairs of cases in unrelated persons in the same household, ≈4*H* pairs of cases would be expected within a village, mostly in different households. If the average village size of ≈138 households estimated for an area of Thailand (*8*) is typical, then if members of all households in a village were at equal risk, we would expect to see far more pairs of unrelated cases within a village than have actually been observed (4*H* ≈550 pairs of cases in unrelated persons, which greatly exceeds the observed 261 total cases). Clearly, this argument is only heuristic, but when this argument is combined with the likelihood of biologic and behavioral differences between households, it seems likely that τ would vary considerably from 1 household to another.

Furthermore, the model does not account for additional individual variability in susceptibility possibly related to age, level of exposure, or other risk factors. If younger persons have a higher risk for infection or likelihood of exposure, clustering would be promoted, primarily within blood relatives, because siblings would be more likely than either parent to become infected. Approximately half of all cases have occurred in those <20 years of age (*9*). Similarly, if female persons (for example) were at higher risk for exposure, infection, or both, then clusters including non–blood relatives (e.g., spouses) would tend to include the low-risk sex and thus be less probable. Female persons of ages 10–29 years were slightly overrepresented among laboratory-confirmed case-patients, but the difference was not statistically significant (*9*).

The null model presented here is not designed to capture all of the heterogeneities in exposure and complexity of real families exposed to influenza subtype H5N1. Rather, it simply illustrates that a large proportion of family clusters limited to blood relatives may occur by chance in the absence of genetic variation in susceptibility, particularly when the probability of infection is low and family sizes are large. Although genetic heterogeneity may possibly contribute to the clustering of avian influenza cases within blood relatives, it is neither a necessary nor the most likely explanation for the data currently available.

## Supplementary Material

Technical Appendix Estimation of the Proportion of Clusters Limited to Blood Relatives
