# Supplementary material for: Little Evidence for Genetic Susceptibility to Influenza A (H5N1) from Family Clustering Data
Source: Emerg Infect Dis. 2007 Jul;13(7):1074–6. doi: 10.3201/eid1307.061538 (PMC2878232; doi:10.3201/eid1307.061538)
Supplement: Technical Appendix — Estimation of the Proportion of Clusters Limited to Blood Relatives [file 06-1538_Techapp-s1.pdf]

Technical Appendix: Supplementary Methods

Estimation of the Proportion of Clusters Limited to Blood Relatives

The expected proportion of clusters limited to blood relatives, p_B, for nuclear families (consisting of two parents and a defined number of children who are the offspring of both parents) who experience a common exposure to an avian influenza virus is given by

$$p_B = 1 - \frac{\tau^2}{1-(1-\tau)^n - n\tau(1-\tau)^{n-1}} . \qquad (1)$$

Here τ^2 is the probability that both parents are infected (hence the cluster is not restricted to blood relatives) and the denominator is the probability that any two or more persons in the household are infected, i.e., the probability of a household cluster. The model can easily be generalized to more complicated family structures.

Formally, let p_B = Pr(BC | C) where BC is the event that a family has a cluster of cases only in blood relatives, and C is the event that the family has a cluster of cases, i.e. more than one case. By the law of conditional probability,

$$p_B = \Pr(BC \mid C) = \frac{\Pr(BC \cap C)}{\Pr(C)} = \frac{\Pr(BC)}{\Pr(C)} = \frac{\Pr(C) - \Pr(NBC)}{\Pr(C)} = 1 - \frac{\Pr(NBC)}{\Pr(C)} \qquad (2)$$

where NBC is the event that a family has a cluster including non-blood relatives: in this case, including both parents. Since a family cluster is defined as two or more cases in a family, Pr(C) = 1 – Pr(0 cases) – Pr(1 case). The simple binomial probabilities of having 0 or 1 case in the family are given by: Pr(0 cases) = $(1 - \tau)^n$ and Pr(1 case) = $n\tau(1 - \tau)^{n-1}$. The probability that the two parents will be infected is just τ^2. Substituting into equation (2) we obtain equation (1).

Accounting for Extended Family Structures

While our null model assumes a nuclear family structure, at least 11 out of the 36 clusters described here include members of the extended family (Appendix Table). We can account for such extended family structures by substituting $u\tau^2$, where u is the number of pairs of unrelated family members, for Pr(NBC) in equation (2) when τ is small, i.e. nearly all clusters are of size 2. Similarly, when τ is small, the denominator also includes only clusters of size 2 and can be approximated as

$$\binom{n}{2}\tau^2 .$$

To take an arbitrary example, if we assume all families have an extended family structure such as the one depicted in Figure S1, there are u = 6 pairs of unrelated family members; therefore, the proportion of clusters limited to blood relatives occurring among such families would be:

$$p_B \approx 1 - \frac{6\tau^2}{\binom{8}{2}\tau^2} = 1 - \frac{6}{28} = \frac{11}{14}.$$

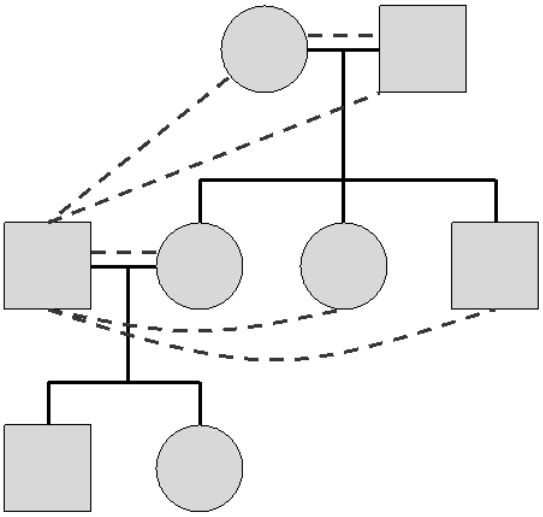

Technical Appendix Figure. Example of an extended family pedigree. There are 8 family members (2 parents, their 3 children, son-in-law, and 2 grandchildren) and 6 pairs of non–blood relatives (depicted by the dashed lines).

Technical Appendix Table. Family clusters of influenza A (H5N1), Dec 2003–Dec 2006*

Cluster	Onset of index case	Country	Age, y/Sex	Relation to index case	H5N1	Onset	Outcome
1	Dec/03	Viet. (N)	12/F	Self	+	Dec 25	D
			30/F	Mother	+	Jan 1	D
2	Dec/03	Viet. (N)	5/M	Self	+	Dec 29†	D
			7/F	Sister	NT	NN	D
3	Jan/04	Viet. (N)	31/M	Self	NT	Jan 7†	D
			30/F	Sister	+	Jan 10	D
			28/F	Wife	+	Jan 10	R
			23/F	Sister	+	Jan 11	D
4	Jan/04	Thailand	6/M	Self	+	Jan 8	D
			33/F	Mother	NT	Jan 8	D
5	Jul/04	Viet. (S)	19/M	Self	NT	Jul 23	D
			22/F	Cousin	NT	NN	D
			25/F	Sister	+	Jul 31	D
6	Sep/04	Thailand	11/F	Self	NT	Sep 2	D
			26/F	Mother	+	Sep 11	D
			32/F	Aunt	+	Sep 16	R
7	Dec/04	Viet. (N)	46/M	Self	+	Dec 26	D
			42/M	Brother	+	Jan 10†	R
			36/M	Brother	+	Not ill	Not ill
8	Jan/05	Viet. (S)	17/M	Self	+	Jan 10†	D
			22/F	Sister	NN	NN	Unk#
9	Jan/05	Viet (S)	35/F	Self	+	Jan 14	D
			13/F	Daughter	+	Jan 20	D
10	Jan/05	Camb.	14/M	Self	NT	NN	D
			25/F	Sister	+	Jan 21	D
11	Feb/05	Viet. (N)	21/M	Self	+	Feb 14	Unk#
			14/F	Sister	+	Feb 23	Unk#
			80/M	Grandfather	+	Not ill	Not ill
12	Feb/05	Viet. (N)	69/M	Self	+	Feb 19	D
			61/F	Wife	+	Not ill	Not ill
13	Mar/05	Viet.	13/F	Self	NT	Mar 9‡	D
			5/M	Brother	+	Mar 12†	R
			Adult/F	Aunt	P	NN	Unk#
14	Mar/05	Viet. (N)	39/M	Self	+	Mar 22†	Unk#
			Adult/F	Wife	+	Mar 22†	Unk#
			4 mo/NN	Child	+	Mar 22†	Unk#
			3/NN	Child	+	Mar 22†	Unk#
			10/NN	Child	+	Mar 22†	Unk#
15	Jul/05	Indonesia	8/F	Self	+	Jun 24	D
			1/F	Sister	NT	Jun 29	D
			38/M	Father	+	Jul 2	D
16	Sep/05	Indonesia	21/M	Self	+	Sep 20	Unk#
			4/M	Nephew	+	Oct 4	R
17	Oct/05	Thailand	48/M	Self	+	Oct 13	D
			7/M	Son	+	Oct 16	Unk#
18	Oct/05	Indonesia	19/F	Self	+	Oct 19	D
			8/M	Brother	+	Oct 25	Unk#
19	Oct/05	China	12/F	Self	NT	16 Oct	D
			9/M	Brother	+	17 Oct	R
20	Nov/05	Indonesia	7/M	Self	NT	3 Nov	D
			20/M	Brother	NT	3 Nov	D
			16/M	Brother	+	6 Nov	Unk#
21	Dec/05	Turkey	14/M	Self	+	26 Dec	D
			15/F	Sister	+	28 Dec	D
			12/F	Sister	+	28 Dec	D
22	Dec/05	Turkey	9/F	Self	+	29 Dec	R
			3/M	Brother	+	30 Dec	R
23	Jan/06	Turkey	5/M	Self	+	8 Jan	R
			14/F	Sister	+	8 Jan	D
24	Jan/06	Indonesia	13/F	Self	+	6 Jan	D
			4/M	Brother	+	8 Jan	D

25	Jan/06	Iraq	15/F	Self	+	17 Jan‡	D
			39/M	Uncle	+	18 Jan	D
26	Feb/06	Azer.	17/F	Self	+	23 Feb‡	D
			20/F	Cousin	+	3 Mar‡	D
			16/M	Cousin	+	10 Mar‡	D
			17/F	Friend	+	8 Mar‡	D
27	Mar/06	Azer.	15/F	Self	+	11 Mar	R
			17/F	Cousin	+	11 Mar	R
28	Mar/06	Egypt	6/F	Self	+	NN	Unk#
			18 mo/F	Sister	+	NN	Unk#
29	Apr/06	Indonesia	37/F	Self	NT	27 Apr	D
			15/M	Son	+	9 May‡	D
			17/M	Son	+	12 May‡	D
			28/F	Sister	+	10 May‡	D
			18 mo/F	Niece	+	14 May‡	D
			25/M	Brother	+	NN	Unk#
			10/M	Nephew	+	13 May‡	D
			32/M	Brother	+	15 May	D
30	May/06	Indonesia	10/F	Self	+	16 May	D
			18/M	Brother	+	16 May	D
31	May/06	Indonesia	15 F	Self	+	17 May	Unk#
			27 M	Brother	+	28 May	R
32	May/06	Indonesia	7/F	Self	+	26 May	D
			10/M	Brother	NT	29 May‡	D
33	Jul/06	Indonesia	17M	Self	+	26 Jul	R
			20M	Cousin	NT	26 Jul	D
34	Sep/06	Indonesia	11M	Self	+	16 Sep	D
			21F	Sister	+	19 Sep	Unk#
35	Sep/06	Indonesia	24 M	Self	NT	16 Sep	D
			20M	Brother	+	17 Sep	D
36	Dec/06	Egypt	30F	Self	+	17 Dec†	D
			16F	Niece	+	19 Dec†	D
			26M	Brother	+	17 Dec†	D

*Camb., Cambodia; D, respiratory death; N, north; NT, not tested; NN, not noted; P, pending; R, recovered; S, south; Unk, unknown; Viet., Vietnam; Azer., Azerbaijan.

†Date of hospitalization.

‡Date of death.

#Had respiratory symptoms, was hospitalized (unknown for #13 and #29), and outcome was unknown.
